# Magnetic field effects on the crystal structure, morphology, energy gap, and magnetic properties of manganese selenide nanoparticles synthesized by hydrothermal method

**DOI:** 10.1039/d3na00730h

**Published:** 2023-10-06

**Authors:** Ali Salmani Nokabadi, Ahmad Yazdani

**Affiliations:** a Department of Condensed Matter Physics, Faculty of Basic Sciences, Tarbiat Modares University Tehran Iran yazdania@modares.ac.ir

## Abstract

In this study, we synthesized manganese selenide under magnetic fields ranging from 0 to 800 gauss and investigated its optical, electrical, and magnetic properties. In the absence of a magnetic field, we observed the formation of MnSe nanorods. As the field strength increased, impurities arose. In the 250 G range, two rock salt structures emerged, altering the morphology from nanorods to cubes. Beyond 250 G, MnSe_2_ formed, returning to a nanorod morphology. Also, with the increase of the magnetic field, the energy gap of the synthesized compounds increased. To measure the electrical properties of the samples, the synthesized powders were compressed under the same pressure for a certain period of time, and it was observed that the synthesized samples showed insulating behavior in the presence of a magnetic field. For this reason, we performed current–voltage, resistance–temperature, and current–temperature analyses on the synthesized sample, at a constant voltage of 5 eV in the absence of a magnetic field.

## Abstract 2

In this study, we synthesized manganese selenide under magnetic fields ranging from 0 to 800 gauss and investigated its optical, electrical, and magnetic properties.

## Graphical abstract

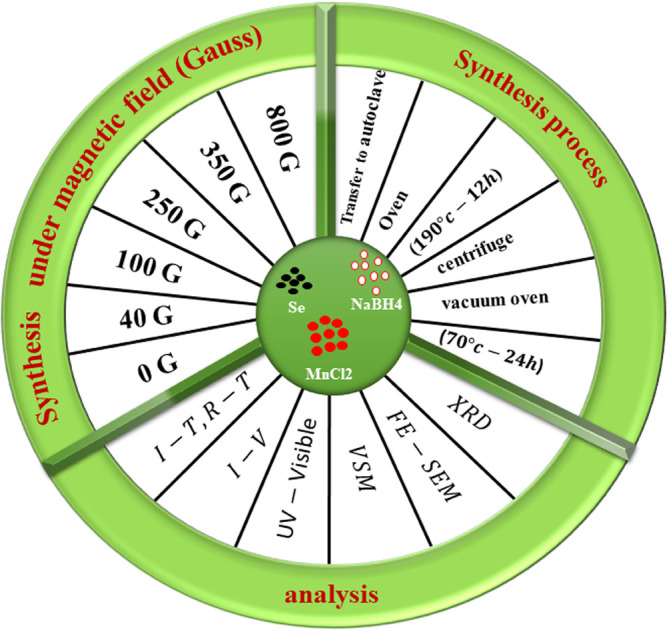

## 1 Introduction

Due to their crucial optical, electrical, and transport characteristics, transition metal chalcogenides (TMC) have caught the interest of researchers, gaining an essential role in superconductors, photocatalysts, semiconductors, supercapacitors, fuel cells, and thermoelectric materials.^1–16^ Also, the conductivity behavior of TMC varies from semiconductor to conductor. Additionally, these compounds have a wide range of magnetic characteristics.^17–23^

The energy difference between valence and conduction bands, known as the band gap, holds significant importance in semiconductor physics. For advancing studies in photovoltaic, photocatalytic, and dye-sensitized solar cell technologies, it is essential to create semiconductor materials with small dimensions and reduced bandgap energies. Semiconductors with wider band gaps can efficiently harness UV and solar light through lower-energy absorptions and excitations. Band gap reduction can occur due to various factors, such as temperature, doping, alloying, pressure, lattice volume, and flaws. However, when studying materials at the nanoscale, it is crucial to characterize the microstructure, focusing on particle size and microstrain. Lattice strain can significantly impact and control the optical, electrical, and mechanical properties of nanoparticles, as it plays a fundamental role in determining their behavior.^24^

One transition metal, manganese, has unique optical and magnetic characteristics that are very important.^25–28^ Researchers have noted its accessibility, affordability, and environmental friendliness, which increase its potential. The compound manganese selenide (MnSe) is a TMC that has captured the interest of many researchers due to its essential magnetic properties. MnSe is a compound that may take on a variety of shapes, such as the wurtzite structure (ZnS),^29^ the rock salt structure (NaCl),^30^ and the hexagonal structure (NiAs).^31^

The rock-salt structure is the most stable form of MnSe at standard room temperatures. The Mn and Se atom arrangements in this crystal structure are alternated on a face-centered cubic (fcc) lattice. The direct bandgap of the semiconductor phase of MnSe in the rock-salt crystal structure is about *E*_g_ = 2 eV.^32^

MnSe may be produced using various techniques, including chemical vapor deposition, solid-state reactions, and procedures that use solutions, such as solvothermal and hydrothermal synthesis.^32,33^ Due to its many benefits over alternative techniques – such as precise control over particle size and shape, high product purity, process scalability, and minimal environmental impact – the hydrothermal method is an effective strategy for synthesizing MnSe.^34^

In previous investigations, researchers have explored various methods for synthesizing manganese selenide. For instance, Decker *et al.*^35^ achieved successful synthesis of α-MnSe by subjecting high-purity Mn metal to elemental Se within a quartz tube at a temperature of 900 °C for 20 h, using a catalyst (I_2_) in the process. They also demonstrated the feasibility of obtaining MnSe_2_ by employing stoichiometric amounts of Mn and Se at a temperature of 550 °C. In solution-phase synthesis, (NH_4_)_2_Se or H_2_Se is commonly used as a selenium source, but these procedures often require advanced equipment, precise reaction conditions, and the use of hazardous and delicate raw materials, which can limit progress in this area. Wu *et al.*,^21^ successfully synthesized manganese selenide utilizing Se powder and a hydrazine reducer, heating the mixture to 1800 °C for 12 h to obtain the desired composition. Similarly, Liu *et al.*,^36^ achieved successful synthesis using SeO_2_ powder and varying concentrations of a hydrazine reducer, with the reaction taking place for 12 h at 1200 °C.

The hydrothermal approach, as documented in previous papers, was applied in this study to create manganese selenide nanorods. Diverse techniques were implemented, encompassing modifications to the reducing agent, adjustments in the synthesis temperature, and variations in the synthesis duration, based on available reports. Ultimately, MnSe nanorods were successfully synthesized using selenium powder, sodium borohydride as the reducing agent, and temperature modifications.^12,37–39^ This research examines how various magnetic fields affect the morphology, energy gap, crystal structure, and magnetic characteristics of MnSe nanoparticles. In order to investigate the impact of magnetic fields on these attributes, MnSe nanoparticles were synthesized under various magnetic field strengths (ranging from 0 to 800 G). This study provides new insights into the interactions between magnetic fields and the characteristics of MnSe nanoparticles, which could have significant implications for spintronics and magnetic storage technologies.

## 2 Experimental

**Fig. 1:**
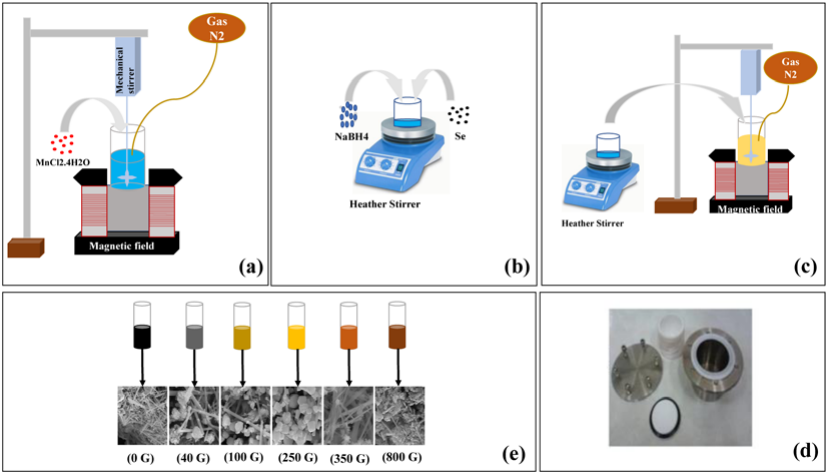
Steps of the synthesis process: (a) adding MnCl_2_·4H_2_O to water under N_2_ gas, (b) adding NaBH_4_ to deionized water and then adding Se to the solution, (c) the solution containing NaBH_4_ and Se is added to the MnCl_2_·4H_2_O solution under N_2_ gas, (d) the solution is poured into the autoclave and placed in the oven, (e) color scheme of synthesized solutions under different magnetic fields.

All chemicals used in this study, including manganese chloride (MnCl_2_·4H_2_O), selenium powder (Se), and sodium borohydride (NaBH_4_), were acquired from Merck Co (>98%) and utilized without additional purification. To prepare manganese selenide nanorods, MnCl_2_·4H_2_O (3 mmol) was dissolved in 50 ml of deionized water, and this solution was placed under N_2_ gas at a pressure of 1 bar for 15 min (Fig. 1a). During the MnCl_2_·4H_2_O test, 6 mmol of the reducing agent NaBH_4_ was poured into 5 ml of deionized water and stirred at 500 rpm for 5 min. After 5 min, selenium powder was added to the NaBH_4_ solution and placed on the stirrer at 600 rpm for 3 min (Fig. 1b). Initially, adding selenium caused the solution to turn black, but after 3 min, it became colorless. The colorless solution was promptly combined with the manganese chloride solution and subjected to a stirring rate of 700 rpm for 10 min under a N_2_ gas atmosphere at a pressure of 1 bar (Fig. 1c). Upon addition of the colorless solution, the overall solution exhibited a creamy appearance. The next phase involved pouring the mixture into the autoclave, which was then heated to 190 °C and left for 12 h (Fig. 1d). The solution was then centrifuged through deionized water and washed with ethanol before being dried in a vacuum oven for 12 h at 70 °C. MnSe nanorods were ultimately produced.

The stages of MnSe synthesis are shown in Fig. 1. These steps were carried out in the presence of an applied magnetic field of 40, 100, 250, 350 or 800 G. The magnetic field was meticulously generated using a pair of precisely calibrated magnetic coils. For example, the MnCl_2_·4H_2_O solution was placed under N_2_ gas in a magnetic field of 40 G. The stage combining NaBH_4_ with Se was not carried out under a magnetic field, but then the resulting colorless solution was added to the manganese solution under the presence of a magnetic field. Finally, after 10 min, we put the solution in an oven. Syntheses carried out under different magnetic fields revealed changes in the color of the synthesized powders with increasing magnetic field strength.

According to the syntheses carried out under different magnetic fields, it was observed that the color of the synthesized powders changes with an increase of the magnetic field. In the synthesis of the original sample, black powder was formed; under a magnetic field of 40 G the powder was grey; under a 100 G magnetic field, a combination of cream and brown appeared; under 250 G, the color became creamier; under 350 G, the color of the powder was a light brown; and under a magnetic field of 800 G, the color of the powder was a burnt brown (Fig. 1e). The dimensions and purity of the resulting materials were assessed utilizing an X-ray diffractometer (XRD) with CuKα radiation (*λ* = 1.54 Å), manufactured by Philips under the XPert MPD model. The morphologies of the materials were investigated using a field-emission scanning electron microscope (FE-SEM), namely a Hitachi S-4160. Ultraviolet-visible (UV-Vis) absorption behavior was recorded with a Unico 4802 UV-vis spectrophotometer spanning the 200–1000 nm range. Magnetic hysteresis loops of the specimens were measured using a vibrating sample magnetometer (VSM) from MDK-Magnetics.

## 3 Results

**Fig. 2:**
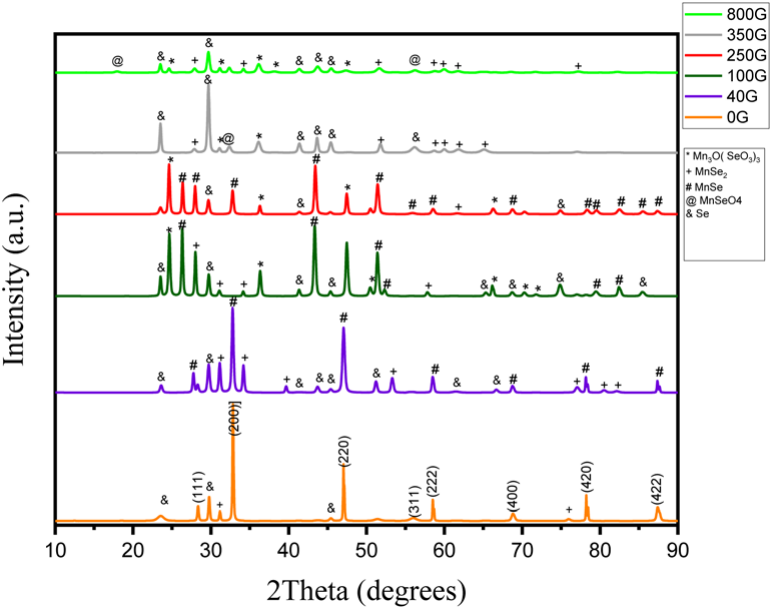
X-ray diffraction of the synthesized samples in the absence of a magnetic field and magnetic fields of 40, 100, 250, 350 and 800 gauss.

The X-ray diffraction patterns of the samples synthesized under different magnetic fields are shown in Fig. 2. In the synthesis without the application of a magnetic field, the peaks are identified as diffraction planes (111), (200), (220), (311), (222), (400), (420), and (422) which are identified by JCPDS card number 00-011-0683 matches and show a cubic structure with lattice constant *a* = 5.64 Å. Of the five impurity peaks shown in the diagram, three are related to selenium impurity, and two are related to MnSe_2_. According to previous articles, this proves the correctness of the synthesis of the α-MnSe compound in the α-MnSe structure.^12,21^

In the sample synthesized under 40 gauss magnetic field, the mold phase is α-MnSe, but the peaks related to Se and MnSe_2_ impurities are increased compared to the synthesis under zero gauss field. Also, the peaks in this synthesized sample correspond to the original sample and the lattice constant remains unchanged.

Under the magnetic field of 100 gauss, the mold phase is α-MnSe with a lattice constant of *a* = 5.90 Å. By increasing the magnetic field during synthesis, the Mn_3_O(SeO_3_)_3_ impurity can also be seen along with Se and MnSe_2_. In addition, the α-MnSe corresponds to the JCPDS card and number 01-088-2344.

Under the field of 250 gauss, the mold phase of α-MnSe is with two lattice constants of *a* = 5.46 Å and *a* = 5.90 Å, which correspond to JCPDS card numbers 01-088-2344 and 00-011-0683. Also, the impurity peaks in this synthesis are the same as the sample synthesized in the magnetic field under 100 gauss.

Under the magnetic field of 350 gauss, with the increase of the magnetic field, the synthesized compound changed to MnSe_2_, and the mold phase is MnSe_2_, which corresponds to the JCPDS card number 00-020-0722, has a cubic structure with a lattice constant of *a* = 6.41 Å. Under the 350 G field, in addition to the impurity peaks that existed in the product under the 100 and 250 G magnetic fields, peaks related to MnSeO_4_ also appeared.

Under the magnetic field of 800 gauss, with the increase of the field, a Mn_3_O(SeO_3_)_3_ impurity is observed along with MnSeO_4_. Also, it is the MnSe_2_ phase, which is compatible with JCPDS card number 01-073-1525, and the lattice constant in this combination is *a* = 6.41 Å.

Finally, the X-ray diffraction patterns of MnSe samples synthesized under the various magnetic fields show changes in the crystal structure and the impurities that are present. In the absence of a magnetic field, the sample exhibited a cubic α-MnSe structure, while under increasing magnetic fields, impurity peaks related to Se and MnSe_2_ appeared, indicating that the magnetic field influenced the formation of impurities in the synthesized samples. The lattice constant of the α-MnSe structure altered as the magnetic field intensity rose, and finally the synthesized substance shifted to the MnSe_2_ phase. The appearance of the Mn_3_O(SeO_3_)_3_ impurity under higher magnetic field strengths, further confirmed the effect of the magnetic field on the synthesis process. All things considered, the findings imply that introducing a magnetic field during synthesis can change the crystal structure and impurity formation in MnSe samples, which may impact their magnetic and electronic characteristics.

The size of the main particles in each sample along with the lattice strain are given in Table 1.

**Table 1:** Crystallite size and lattice strain of the synthesised samples in the absence and presence of a magnetic field

Sample synthesized	0 G	40 G	100 G	250 G	350 G	800 G
Crystallite size [Å]	863	323	378	301	262	262
Lattice strain [%]	0.142	0.38	0.403	0.415	0.494	0.494
Average particle size [nm]	242.91	90.9	156.49	316.68	97.95	75.20

The magnetic field impacts the crystal lattice during the synthesis process, as the magnetic field causes a change in the lattice strain. The lattice strain rises as a function of the magnetic field’s intensity since it can result in a more considerable degree of lattice distortion. Furthermore, its interaction with atomic magnetic moments could generate stresses within the crystal lattice. The occurrence of lattice strain might also be affected by the presence of defects or impurities induced by the field. Additionally, the size reduction of the crystals with an increase in the magnetic field can be attributed to the field applied to the particles during the synthesis process, which could impede nucleation and result in smaller crystallite sizes. The magnetic field might play a crucial role in the alignment and aggregation of particles in the solution, enhancing nucleation efficiency and promoting the growth of smaller particles. Moreover, the magnetic field may influence the diffusion and transport of reactants, leading to more uniform nucleation and smaller crystallite sizes. In essence, the magnetic field can be employed as an adjustable parameter to control the size of the crystals during synthesis.

**Fig. 3:**
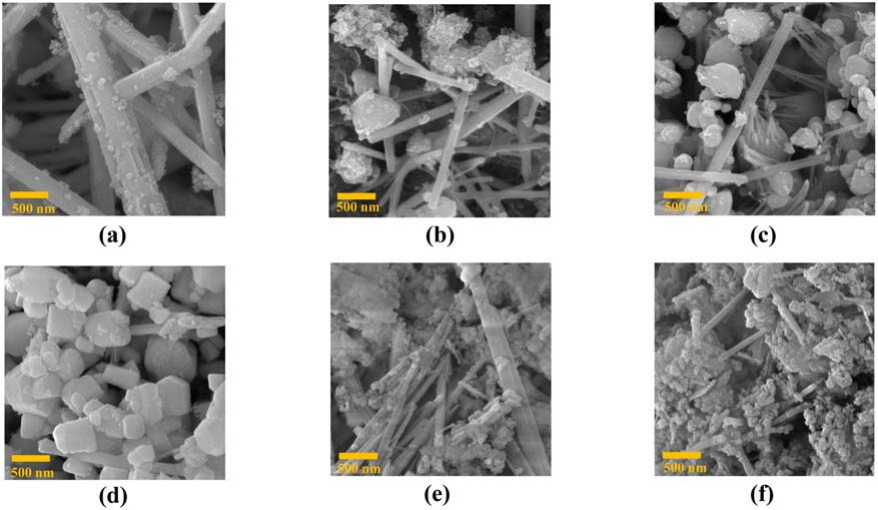
FE-SEM analysis in the 500 nm range: (a) sample synthesized without applying magnetic field, (b) sample synthesized under 40 G magnetic field, (c) sample synthesized under 100 G magnetic field, (d) synthesized sample under 250 G magnetic field, (e) the sample synthesized under 350 G magnetic field, and (f) the sample synthesized under the 800 G magnetic field.

As shown in Fig. 3, our results clearly demonstrate the significant impact of the magnetic field on the final product’s appearance. Crystals with a nanorod shape form when there is no magnetic influence. However, as the intensity of the magnetic field increases, it starts affecting crystal growth, causing the crystals to grow at unexpected orientations. Consequently, the number of nanorods decreases, and the crystals transform into ‘sugar’ cubes.

As the magnetic field intensity continues to rise above 250 G, the cube crystals disappear, and the nanorods reappear, indicating a shift in the direction of crystal growth. The presence of impurities in the morphology influences the crystal growth process, leading to changes in the crystal shape and size. All these findings highlight the crucial role the magnetic field plays in determining the crystal structure and morphology of the synthesized sample.

**Fig. 4:**
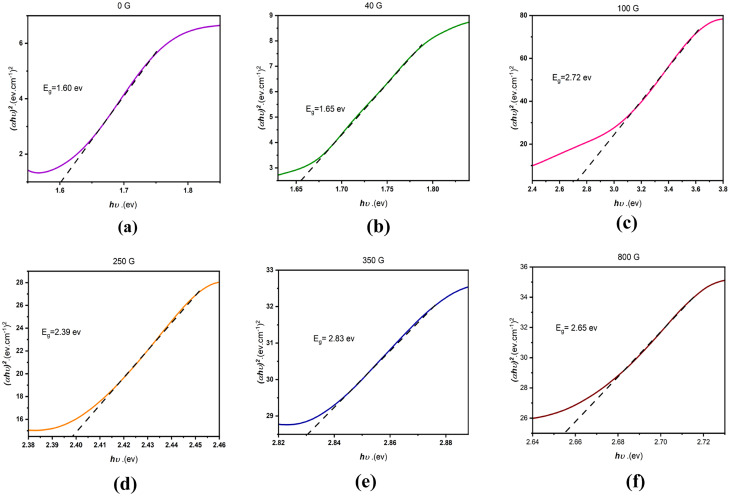
Energy gap diagrams drawn by the tack plot method. (a) Sample synthesized without applying a magnetic field, (b) sample synthesized under the application of a 40 G magnetic field, (c) sample synthesized under the application of a 100 G magnetic field, (d) sample synthesized under the application of a 250 G magnetic field, (e) sample synthesized under the application of 350 G magnetic field and (f) sample synthesized under the application of an 800 G magnetic field.

The results presented in Fig. 4 display variations in the energy gap for the synthesized samples. The energy gap shows an upward trend as the magnetic field increases. It is well-known that the application of a magnetic field impacts electron spin and orbital motion, resulting in modifications to the band structure and density of states. In the specific case of MnSe, higher magnetic fields might reduce the overlap between Se (4p) and Mn (3d) orbitals, consequently leading to an increase in the energy gap.^40^ This effect tends to be more prominent at lower magnetic fields, where the orbital overlap is stronger in the absence of a magnetic field.

The decrease in the energy gap at high magnetic fields (800 gauss) could be due to the onset of magnetic saturation in the material, which can lead to a reduction in the magnetic moment and a weakening of the effect of the magnetic field on the electron structure. Additionally, the presence of impurity phases at higher magnetic fields could also contribute to the observed changes in the energy gap.

The subsequent steps in our study involved subjecting all synthesized samples to a uniform pressure of 50 kg m^−2^ for a duration of 24 h. This procedure was crucial for conducting thorough analyses of current–voltage characteristics, temperature–current relationships, and resistance–temperature behaviors. Notably, all samples, with the exception of the one synthesized without applying a magnetic field, displayed insulating behavior. This observation prompted us to investigate the FE-SEM analysis of all samples. It is worth noting that discontinuities between the crystals of these samples, which show insulating behavior, could be detected.

**Fig. 5:**
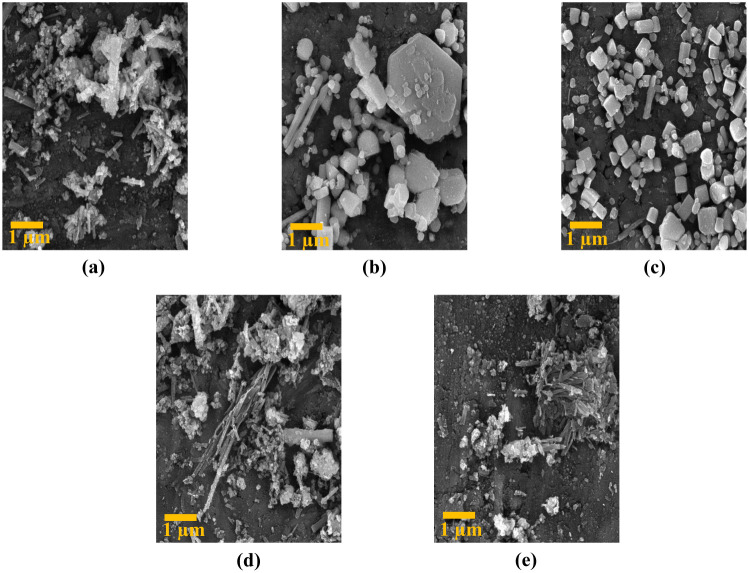
FE-SEM analysis at 1 μm scale: (a) sample synthesized under 40 G magnetic field, (b) sample synthesized under 100 G magnetic field, (c) sample synthesized under 250 G magnetic field, (d) sample synthesized under 350 G magnetic field, and (e) sample synthesized under 800 G magnetic field.

This finding shows that the insulating behavior observed in the samples can be attributed to the presence of physical discontinuities or fractures in the crystal structure. Such breaks could disrupt the efficient movement of charge carriers, leading to the observed insulating characteristics (Fig. 5).

**Fig. 6:**
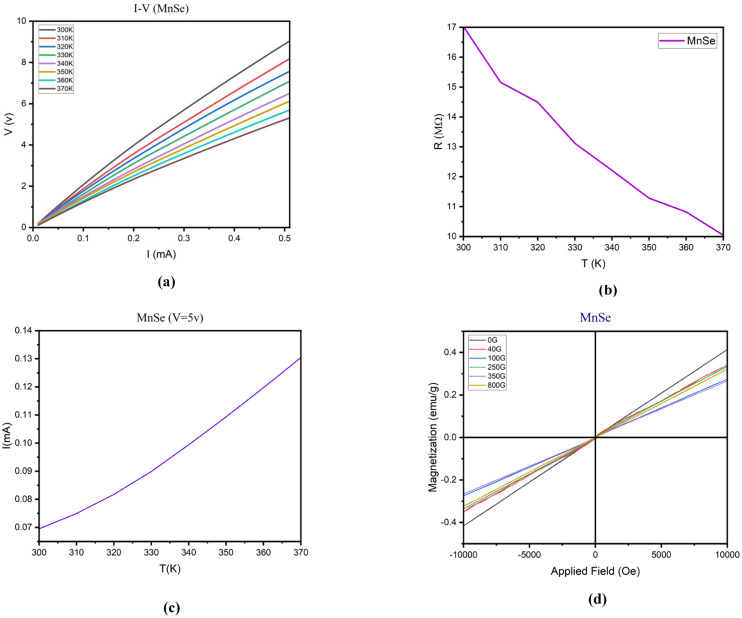
Analyses of (a) current–voltage, (b) resistance–temperature, (c) current–temperature at a constant voltage of 5 V and (d) magnetization.

According to Fig. 6:

(a) the exponential increase in current in the low voltage range (0.01–0.1 mA) is likely due to the presence of surface states in the MnSe tablet. These surface states can act as traps for charge carriers, leading to a non-linear response to voltage. As the voltage increases, more charge carriers can overcome these traps and contribute to the current, resulting in an exponential increase in current. The gradual rise in current observed in the upper voltage range (0.1–0.5 mA) is probably attributed to the overall conductivity of the MnSe sample. As the voltage steadily escalates, an increasing number of charge carriers are enabled to partake in the current flow, leading to a linear augmentation in current.

(b) The reduction in resistance as the temperature rises is a widely observed occurrence in most materials and is known as the positive temperature coefficient of resistance (PTCR) phenomenon. The PTCR effect arises due to the thermal activation of charge carriers, leading to an increase in the number of charge carriers available for conduction and, consequently, a decline in resistance.^41^

However, within the temperature range 310–320 K, the slope of the resistance–temperature curve differs from that of other temperature ranges. This indicates that factors beyond the thermal activation of charge carriers influence the resistance within this specific temperature interval.

One possible reason for this behavior is that the sample undergoes a structural alteration within this temperature range. Such a transformation might trigger modifications in the crystal structure, lattice parameters, or defect concentration, thereby affecting the material’s electronic and transport properties.

Another possible explanation is that the sample experiences a phase transition within this temperature range, leading to changes in the material’s electronic and transport properties. For instance, this phase transition could bring about alterations in the number or mobility of charge carriers, subsequently impacting the resistance.

(c) The increase in current with temperature can be explained by the increase in thermal energy, which leads to the excitation of more electrons and their promotion to the conduction band. In the range 300–320 K, the increase in current is exponential because the thermal energy is not yet sufficient to overcome the bandgap energy, and only a small number of electrons can be excited to the conduction band. However, as the temperature increases beyond 320 K, the thermal energy becomes sufficient to overcome the band gap energy, and a larger number of electrons can be excited to the conduction band, leading to a linear increase in current.

(d) We observe a clear trend of declining magnetization from the sample synthesized with the lowest magnetic field of 40 gauss to the one with the highest magnetic field of 800 gauss. This pattern can be explained by considering the magnetic characteristics of the material and the influence of external magnetic fields during the synthesis process.

When a magnetic material is exposed to an external magnetic field, its magnetic domains align with the applied field, resulting in an increase in magnetization. However, upon removing the external field, the magnetic domains tend to disperse randomly, potentially causing a partial or complete loss of magnetization. This phenomenon is referred to as magnetic hysteresis.

Regarding the MnSe sample, the original specimen, synthesized without the application of any external magnetic field, displays the highest magnetization because it was not affected by any external field that could disturb its magnetic domains. Conversely, the samples synthesized with progressively increasing magnetic fields (ranging from 40 to 800 gauss) encountered progressively stronger external fields during synthesis, which might have disrupted their magnetic domains, leading to lower magnetization values.

It is also worth mentioning that the antiferromagnetic phase observed in all samples indicates that the magnetic moments of Mn and Se ions are oppositely aligned, resulting in a net magnetization of zero. However, an external magnetic field can induce a nonzero magnetization by breaking the antiferromagnetic alignment, leading to a net magnetic moment in the material. The decreasing trend of magnetization with increasing external field strength suggests that the external field was not potent enough to fully align the magnetic moments in the material.

## 4 Conclusions

In this research, we investigated the synthesis of manganese selenide nanorods under a range of magnetic fields (0–800 gauss). X-ray diffraction revealed changes in the crystal structure and impurities with increasing field strength, impacting crystal growth and the energy gap. The magnetization of the samples decreased with stronger magnetic fields, indicating the disruption of magnetic domains. For the synthesized sample without a magnetic field, the resistance–temperature curve exhibited a positive temperature coefficient of resistance (PTCR) effect. Magnetic fields play a vital role in controlling the crystal structure, impurities, and magnetic properties of MnSe samples.

## Author contributions

Dr Ahmad Yazdani came up with the idea and oversaw the project. Ali Salmani Nokabadi carried out all synthesis steps and measurements and wrote the manuscript.

## Conflicts of interest

There are no conflicts to declare.

## Supplementary Material
